# Molecular Mapping of Flowering Time Major Genes and QTLs in Chickpea (*Cicer arietinum* L.)

**DOI:** 10.3389/fpls.2017.01140

**Published:** 2017-07-06

**Authors:** Bingi P. Mallikarjuna, Srinivasan Samineni, Mahendar Thudi, Sobhan B. Sajja, Aamir W. Khan, Ayyanagowda Patil, Kannalli P. Viswanatha, Rajeev K. Varshney, Pooran M. Gaur

**Affiliations:** ^1^International Crops Research Institute for the Semi-Arid TropicsPatancheru, India; ^2^Department of Genetics and Plant Breeding, University of Agricultural SciencesRaichur, India; ^3^The UWA Institute of Agriculture, University of Western AustraliaPerth, WA, Australia

**Keywords:** earliness, flowering time, chickpea, consensus map, QTLs

## Abstract

Flowering time is an important trait for adaptation and productivity of chickpea in the arid and the semi-arid environments. This study was conducted for molecular mapping of genes/quantitative trait loci (QTLs) controlling flowering time in chickpea using F_2_ populations derived from four crosses (ICCV 96029 × CDC Frontier, ICC 5810 × CDC Frontier, BGD 132 × CDC Frontier and ICC 16641 × CDC Frontier). Genetic studies revealed monogenic control of flowering time in the crosses ICCV 96029 × CDC Frontier, BGD 132 × CDC Frontier and ICC 16641 × CDC Frontier, while digenic control with complementary gene action in ICC 5810 × CDC Frontier. The intraspecific genetic maps developed from these crosses consisted 75, 75, 68 and 67 markers spanning 248.8 cM, 331.4 cM, 311.1 cM and 385.1 cM, respectively. A consensus map spanning 363.8 cM with 109 loci was constructed by integrating four genetic maps. Major QTLs corresponding to flowering time genes *efl-1* from ICCV 96029, *efl-3* from BGD 132 and *efl-4* from ICC 16641 were mapped on CaLG04, CaLG08 and CaLG06, respectively. The QTLs and linked markers identified in this study can be used in marker-assisted breeding for developing early maturing chickpea.

## Introduction

Chickpea (*Cicer arietinum* L.) is a diploid annual legume with 2*n* = 16 chromosomes and a genome size of 738 Mb (Varshney et al., 2013). It is the world's second most important pulse crop after common bean with a total annual production of 13 million tons from an area of 13 million hectares (FAOSTAT, 2015). India, the largest producer and also the largest consumer of chickpeas in the world has 71% of global chickpea area.

Chickpea is a cool season crop mostly cultivated on residual soil moisture in the post-rainy season of the arid and semi-arid regions. Thus, the crop grows and matures on a progressively depleting soil moisture and experiences terminal drought (Kumar and Abbo, 2001). Terminal drought has become a major constraint in many chickpea growing areas. In addition, a large shift in chickpea area from cooler long-season environments to warmer short-season environments has increased the chances of exposure of crop to moisture and heat stresses at the reproductive stage causing severe yield losses (Gaur et al., 2014). Early maturity has been identified as an important trait for increasing and stabilizing chickpea productivity by avoiding end of season drought (Subbarao et al., 1995; Kumar and Abbo, 2001) and frost (Warkentin et al., 2003) in short season environments. Significant impact of early maturing chickpea varieties in horizontal expansion of the crop in the semi-arid tropics has been reported (Than et al., 2007; Gaur et al., 2008).

Flowering time plays a key role in adaption and yield stabilization. It can be recorded with high precision and provides a good indication of succeeding phenological traits such as time of podding and maturity (Gaur et al., 2015). Large genotypic variations exist for flowering time in chickpea. Flowering time is a highly variable trait affected by various factors like soil moisture, photoperiod, temperature, altitude and latitude. The information available on genetics of flowering time in chickpea suggests that flowering time is controlled by one or a few major genes (Or et al., 1999; Kumar and van Rheenen, 2000; Anbessa et al., 2006; Hegde, 2010; Gaur et al., 2015; Gumber and Sarvjeet, 1996). Four flowering time genes have so far been identified in chickpea: *efl-1* from ICCV 2 (Kumar and van Rheenen, 2000), *efl-2* or *ppd* from ICC 5810 (Or et al., 1999), *efl-3* from BGD 132 (Hegde, 2010) and *efl-4* from ICC 16641 (Gaur et al., 2015). Studies have shown that these flowering time genes are non-allelic (Hegde, 2010; Gaur et al., 2015).

Studies have been conducted on molecular mapping of genes/quantitative trait loci (QTLs) controlling flowering time in chickpea. The major gene from ICCV 2 (*efl-1*) was mapped on LG03 (Cho et al., 2002; Jamalabadi et al., 2013). Recently Daba et al. (2016) identified QTLs for days to flowering from ICCV 96029 on LG03, LG04, LG05 and LG08. The major QTLs from ICC 5810 were mapped on LG01, LG02 and LG08 (Lichtenzveig et al., 2006). The flowering time genes *efl-3* (from BGD 132) and *efl-4* (from ICC 16641) are not yet mapped. Several other studies also reported QTLs for flowering time on LG01 (Rehman et al., 2011), LG02 (Karami et al., 2015), LG03 (Cobos et al., 2009; Aryamanesh et al., 2010; Hossain et al., 2010; Rehman et al., 2011; Karami et al., 2015; Upadhyaya et al., 2015), LG04 (Cobos et al., 2007; Varshney et al., 2014; Upadhyaya et al., 2015, LG05 (Upadhyaya et al., 2015) and LG08 (Rehman et al., 2011; Varshney et al., 2014) using different parental lines in chickpea. Identification of QTLs on different linkage groups shows that genes governing flowering time are distributed throughout the genome. Therefore, identification of specific genomic regions controlling different earliness genes assumes greater significance in chickpea improvement. The present study was carried out primarily to identify the genomic regions controlling flowering time genes using four F_2_ populations derived from the crosses ICCV 96029 × CDC Frontier, ICC 5810 × CDC Frontier, BGD 132 × CDC Frontier and ICC 16641 × CDC Frontier.

## Materials and methods

### Plant material

Four early flowering lines ICCV 96029, ICC 5810, BGD 132 and ICC 16641 were crossed to a late flowering cultivar CDC Frontier. The early flowering lines chosen represent different sources of earliness genes based on the previous studies (Or et al., 1999; Kumar and van Rheenen, 2000; Hegde, 2010; Gaur et al., 2015). CDC Frontier is an improved kabuli cultivar with medium maturity developed at the University of Saskatchewan (Warkentin et al., 2005) and the genome sequence of this line was deciphered recently (Varshney et al., 2013). The F_1_ seeds from all the crosses were selfed to develop F_2_ mapping populations. The F_2_ populations along with their parents and F_1_s were evaluated for segregation of flowering time during post rainy season of 2013–14 at International Crops Research Institute for the Semi-Arid Tropics (ICRISAT), Patancheru, Telangana, India. A total of 190 F_2_ genotypes each of the crosses ICCV 96029 × CDC Frontier, ICC 5810 × CDC Frontier, BGD 132 × CDC Frontier and 146 F_2_s of the cross ICC 16641 × CDC Frontier were evaluated. F_3_ progenies with 20 plants in each progeny row were evaluated for flowering time during post rainy season of 2014–15 with 164, 174, 182 and 102 progeny rows from each cross, respectively.

### Phenotyping and statistical analysis

Flowering time was recorded as number of days from seeding to appearance of first fully opened flower on parents, F_1_s and F_2_ populations. In F_3_, each progeny row was observed for flowering time at regular intervals and classified them as non-segregating and segregating types. Flowering time data was used to estimate the parameters of descriptive statistics and segregation analysis (Microsoft Excel, 2013). The expected values corresponding to the observed values for late and early flowering plants were calculated on the basis of the assumed Mendelian ratio. The deviations of these values were subjected to chi-square test to determine the goodness of fit.

### Genomic DNA extraction and marker genotyping

Young leaf tissues from 20 days old seedlings of parents and F_2_ individuals were collected. Extraction of genomic DNA was carried out following high-throughput mini DNA extraction protocol as reported by Cuc et al. (2008). A total of 472 SSR markers reported earlier were used for parental polymorphism screening (including 146 CaM-series markers, Thudi et al., 2011; 124 ICCM markers, Nayak et al., 2010; 135 SSRs, Winter et al., 1999; 57 H-series markers, Lichtenzveig et al., 2005; and 10 NCPGR markers, Sethy et al., 2006; Gaur et al., 2011). SSR (CaM-series, ICCM-series, H-series, Winter-series and NCPGR markers) genotyping, PCR amplification, separation and visualization of amplified products were carried out by following the method described in previous studies (Nayak et al., 2010; Thudi et al., 2011).

### Construction of genetic linkage map and QTL analysis

Genotypic data of all polymorphic markers from four mapping populations were compiled and linkage analysis was performed separately using JoinMap version 4.0 software (Van Ooijen, 2006) as described by Bohra et al. (2012). A consensus map was constructed based on data sets from four populations using JoinMap 4.0 software.

QTL analysis of flowering time was carried out employing inclusive composite interval mapping (ICIM) using QTL-ICiMapping software version 4.0 (Wang et al., 2014). ICIM-Add mapping performs a stepwise regression to identify the most significant markers and marker-pair multiplications at 0.001 probability level and then scanning step of 1 cM. Later, a one-dimensional scanning or interval mapping was conducted to identify additive QTLs. The threshold levels to declare significance of a QTL was determined by performing 1,000 permutations by maintaining the chromosome-wise type I error rate of 0.05 (Churchill and Doerge, 1994). The LOD score peaks were used to estimate the most likely position of a QTL on the linkage map. The amount of variation explained by marker was determined using the coefficient of determination (*R*^2^) value and expressed as percent phenotypic variance explained (PVE%). In this study, a QTL that explains more than 10% of total PVE was considered as major QTL.

## Results

### Genetics of flowering time genes

#### Flowering time of parental lines, F_1_s and F_2_ populations

**Figure 1:**
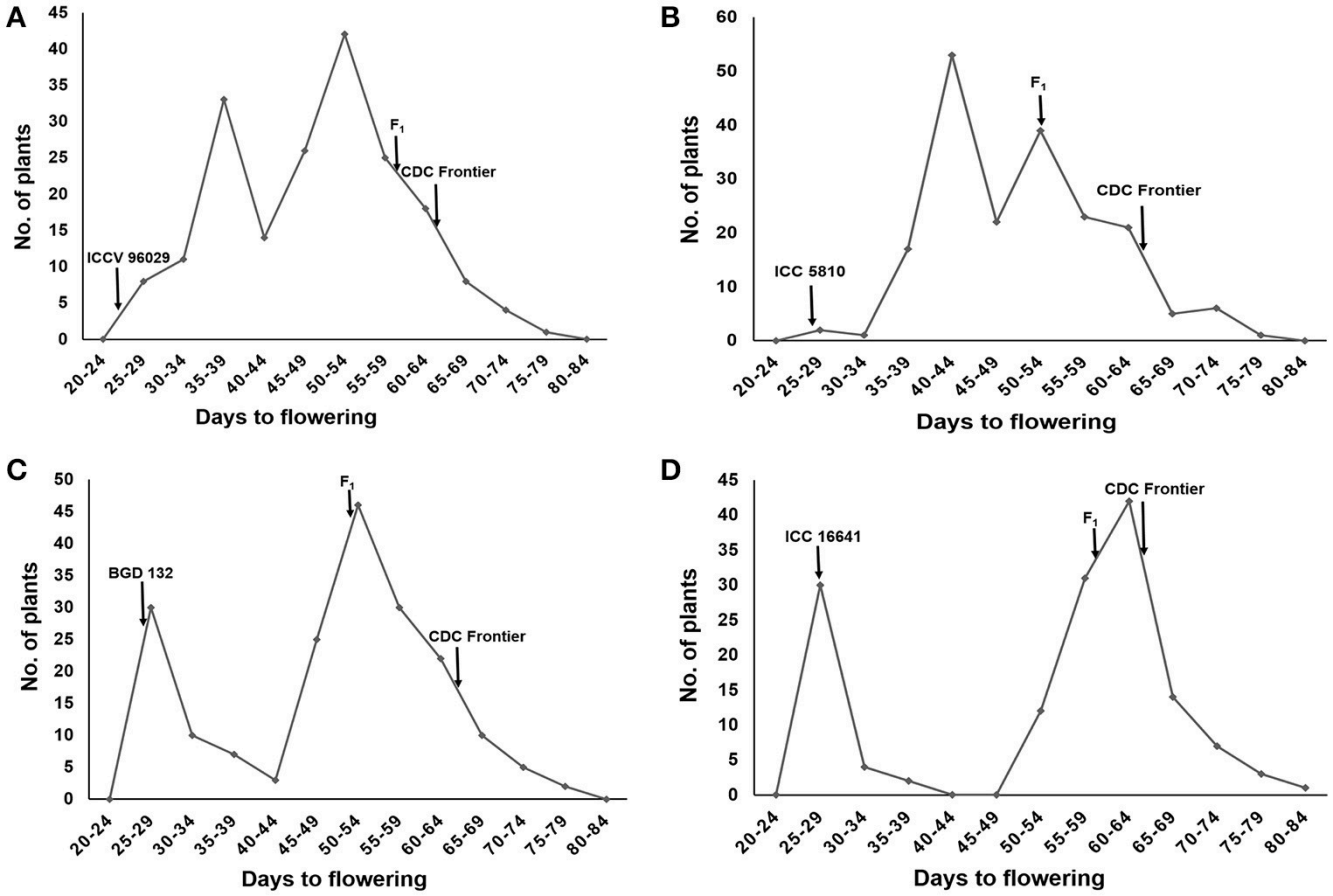
Frequency distribution of flowering time in F_2_s of different crosses: **(A)** ICCV 96029 × CDC Frontier **(B)** ICC 5810 × CDC Frontier **(C)** BGD 132 × CDC Frontier **(D)** ICC 16641 × CDC Frontier.

The female parents ICCV 96029, ICC 5810, BGD 132 and ICC 16641 started flowering at 25, 28, 28 and 29 days after sowing, respectively. Whereas, the male parent CDC frontier flowered at 65 days (Supplementary Table 1). The F_1_s of all the crosses flowered late with mean flowering time of 61.2 (ICCV 96029 × CDC Frontier), 54.1 (ICC 5810 × CDC Frontier), 53.3 (BGD 132 × CDC Frontier) and 60.8 (ICC 16641 × CDC Frontier) days (Supplementary Table 2). A high range was observed for flowering time in F_2_s and the bimodal distribution of flowering time data with unequal peaks (Figures 1A–D) facilitated classification of phenotypic data into early and late flowering types in all the crosses.

#### Segregation of flowering time genes in F_2_

With the classification of the flowering time data into two classes–early (40 days or earlier) and late (later than 40 days), 190 F_2_ plants of the cross ICCV 96029 × CDC Frontier segregated into 138 late and 52 early flowering types (Table 1). These numbers are in good fit with the expected ratio of 3 late: 1 early (χ^2^ = 0.57, *P* = 0.5—0.3). In BGD 132 × CDC Frontier, 190 F_2_ individuals fell into 3:1 ratio with 143 late: 47 early flowering plants (χ^2^ = 0.01, *P* = 0.95–0.9). Similarly, 146 F_2_ plants segregated into 110 late and 36 early flowering plants in ICC 16641 × CDC Frontier. This ratio is in good fit with expected 3:1 ratio (χ^2^ = 0.01, *P* = 0.95–0.9). Whereas, 190 F_2_ individuals segregated into 108 late (later than 45 days) and 82 early (45 days or earlier) in ICC 5810 × CDC Frontier which is in good fit with expected ratio of 9:7 (χ^2^ = 0.03, *P* = 0.9–0.8).

**Table 1:** Segregation of flowering time in F_2_ of four chickpea crosses.

**Sl. No**	**Cross**	**N**	**Observed**		**Expected**		**Ratio tested**	**χ^2^**	*P*-value^*^
			**Late**	**Early**	**Late**	**Early**			
1	ICCV 96029 × CDC Frontier	190	138	52	142.5	47.5	3:1	0.57	0.5–0.3
2	ICC 5810 × CDC Frontier	190	108	82	106.8	83.1	9:7	0.03	0.9–0.8
3	BGD 132 × CDC Frontier	190	143	47	142.5	47.5	3:1	0.01	0.95–0.9
4	ICC 16641 × CDC Frontier	146	110	36	109.5	36.5	3:1	0.01	0.95–0.9

* *Null hypothesis of the test is that progeny segregate in the ratios tested. If the p-value (probability) is less than or equal to 0.05, then reject the null hypothesis. Otherwise one fails to reject the null hypothesis*.

#### Segregation of flowering time genes in F_3_

A total of 164, 174, 182 and 102 F_2_-derived F_3_ families were evaluated for flowering time (Supplementary Table 3). In ICCV 96029 × CDC Frontier, all the 37 early flowering F_2_ plants did not segregate in F_3_ (χ^2^ = 0, *P* = 1.0). Of the 127 late flowering plants, 87 segregated into late and early flowering progenies, remaining 40 showed no segregation and produced only late flowering plants (χ^2^ = 0.19, *P* = 0.7–0.5) in F_3_. In BGD 132 × CDC Frontier also, all the 44 early flowering F_2_ plants did not segregate in F_3_ (χ^2^ = 0, *P* = 1.0). While 94 out of 138 late flowering F_2_ plants segregated into late and early flowering plants and remaining 44 progenies produced only late flowering plants (χ^2^ = 0.13, *P* = 0.8–0.7). Similarly in ICC 16641 × CDC Frontier, all the 25 early flowering F_2_ plants produced only early flowering progenies in F_3_ (χ^2^ = 0, *P* = 1.0). Of the 77 late flowering F_2_ plants, 54 segregated into early and late flowering progenies and remaining 23 progeny lines produced uniform late flowering progenies (χ^2^ = 0.41, *P* = 0.7–0.5). In ICC 5810 × CDC Frontier, 27 out of 71 early flowering F_2_ plants produced only early flowering progenies in F_3_, and remaining 44 segregated into early and late flowering progenies (χ^2^ = 0.67, *P* = 0.5–0.3). Whereas, 103 late flowering F_2_ plants produced 16 F_3_ progenies with uniform late flowering and remaining 87 segregated to produce late and early flowering progenies (χ^2^ = 2.04, *P* = 0.2–0.1) in F_3_. These results gave conclusive evidence of major gene inheritance of flowering time genes in these crosses.

#### Individual genetic maps and consensus map

Among 472 SSRs tested, 100, 95, 90 and 93 were found polymorphic between the parents of the crosses ICCV 96029 × CDC Frontier, ICC 5810 × CDC Frontier, BGD 132 × CDC Frontier and ICC 16641 × CDC Frontier, respectively (Supplementary Table 4). Based on the distribution of markers on chickpea genome, a total of 76, 77, 68 and 68 polymorphic SSR markers were genotyped on the respective mapping population and then used for linkage map construction for each population separately. As a result, genetic linkage map for each cross and a consensus map were developed (http://cegresources.icrisat.org/cmap/sm/cp/mallikarjuna/) and the details are given below.

#### ICCV 96029 × CDC frontier

A total of 75 SSR marker loci were mapped on 8 linkage groups (CaLGs) having a total map length of 248.76 cM and an average inter-marker distance of 3.32 cM (Table 2, Supplementary Figure 1). One marker (TA76s) remained unlinked to any of the linkage groups.

**Table 2:** Features of four intra-specific genetic maps and consensus map.

	**ICCV 96029** × **CDC Frontier**			**ICC 5810** × **CDC Frontier**			**BGD 132** × **CDC Frontier**			**ICC 16641** × **CDC Frontier**			**No. of common markers among four crosses**	**Consensus map**		
**Linkage group**	**No. of markers**	**Map length (cM)**	**Inter-marker distance (cM)**	**No. of markers**	**Map length (cM)**	**Inter-marker distance (cM)**	**No. of markers**	**Map length (cM)**	**Inter-marker distance (cM)**	**No. of markers**	**Map length (cM)**	**Inter-marker distance (cM)**		**No. of markers**	**Map length (cM)**	**Inter-marker distance (cM)**
CaLG01	5	5.85	1.17	6	28.33	4.72	4	32.76	8.19	5	41.05	8.21	1	8	32.04	4.01
CaLG02	7	6.03	0.86	9	29.18	3.24	8	10.39	1.30	5	41.77	8.35	1	11	39.91	3.63
CaLG03	12	43.69	3.64	13	40.88	3.14	15	80.12	5.34	14	58.65	4.19	5	22	61.65	2.80
CaLG04	11	43.47	3.95	13	84.50	6.50	9	45.17	5.02	13	74.75	5.75	5	17	78.75	4.63
CaLG05	10	5.46	0.55	6	6.74	1.12	8	7.88	0.99	9	10.74	1.19	4	13	6.94	0.53
CaLG06	13	72.02	5.54	12	76.28	3.36	9	65.36	7.26	8	65.58	8.20	4	16	73.3	4.58
CaLG07	12	27.02	2.25	11	8.84	0.80	10	37.59	3.76	8	46.32	5.79	6	13	33.87	2.61
CaLG08	5	45.22	9.04	5	56.62	11.32	5	31.83	6.37	5	46.26	9.25	2	9	37.39	4.15
Total	75	248.76	3.32	75	331.37	4.42	68	311.10	4.58	67	385.13	5.75	28	109	363.85	3.34

#### ICC 5810 × CDC frontier

The genetic linkage map consisted of 75 SSR marker loci that are distributed across 8 linkage groups spanning 331.37 cM with an average marker density of one marker per 4.42 cM (Table 2, Supplementary Figure 2). Two markers (TA93 and TA76s) remained unlinked to any of the linkage groups.

#### BGD 132 × CDC frontier

A total of 68 SSR marker loci were mapped on 8 linkage groups having a total map length of 311.10 cM and an average inter-marker distance of 4.58 cM (Table 2, Supplementary Figure 3). No marker was found unlinked after linkage group assignment and ordering.

#### ICC 16641 × CDC frontier

The intraspecific linkage map of this cross consisted of 67 SSR markers mapped onto 8 linkage groups spanning a total map length of 385.13 cM with an average marker density of 5.75 cM (Table 2, Supplementary Figure 4). Only one marker i.e., TA93 was unassigned to any of the linkage groups.

**Figure 2:**
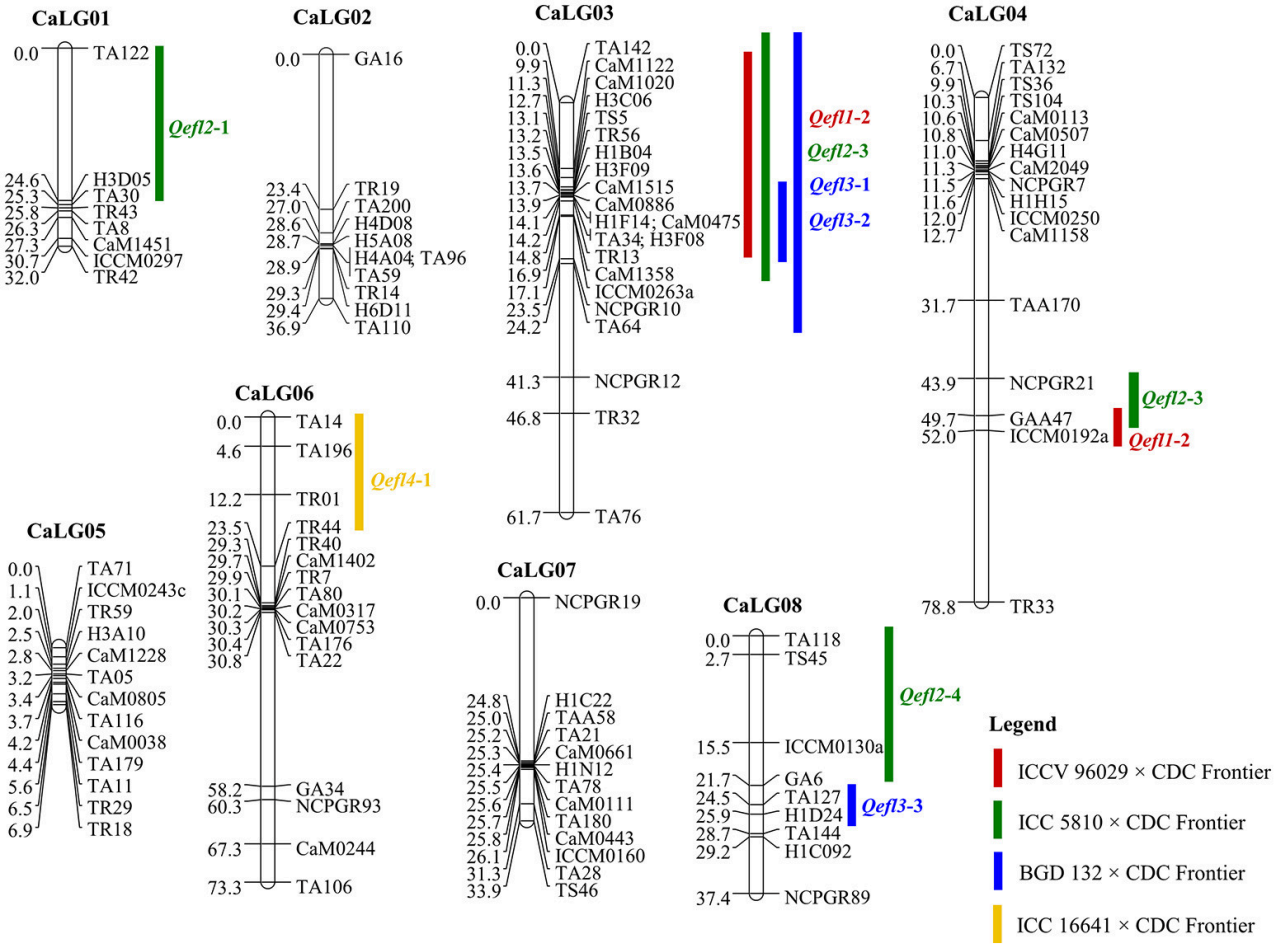
Consensus genetic map comprising 109 marker loci based on four intra-specific mapping populations. Markers are shown on right side of the linkage group while map distances are shown on the left side. The QTLs identified for flowering time in the crosses ICCV 96029 × CDC Frontier, ICC 5810 × CDC Frontier, BGD 132 × CDC Frontier and ICC 16641 × CDC Frontier populations are shown here.

Four genetic maps were integrated using JoinMap 4.0 to develop a consensus map that comprised of 8 linkage groups containing 109 markers with a total map length of 363.85 cM (Table 2, Figure 2). The map lengths of linkage groups in the consensus map were 32.04, 39.91, 61.65, 78.75, 6.94, 73.3, 33.87 and 37.39 cM for CaLG01, CaLG02, CaLG03, CaLG04, CaLG05, CaLG06, CaLG07 and CaLG08 with 8, 11, 22, 17, 13, 16, 13 and 9 marker loci, respectively. The average density of the consensus map was 3.34 cM per marker.

#### QTLs for flowering time genes

Ten significant QTLs were identified for flowering time in this study using QTL-ADD model of the ICIM software. The details of QTLs identified in each cross are presented below:

#### ICCV 96029 × CDC frontier

A major QTL “*Qefl1*-2” was identified for flowering time on CaLG04 in the marker interval GAA47-ICCM192a with a peak LOD value of 5.66 and PVE of 11.75% (Table 3, Supplementary Table 6, and Supplementary Figures 1, 5). In addition, a minor QTL “*Qefl1*-1” was also identified on CaLG03 in the marker interval CaM1122-TR13 with a peak LOD value of 3.45 and PVE of 5.66%. Both the QTLs showed negative additive effect indicating that allele for early flowering at this locus was contributed by ICCV 96029.

**Table 3:** QTLs identified for flowering time in four chickpea crosses.

**Sl. No**.	**Cross**	**QTL**	**CaLG**	**Position (cM)**	**LOD**	**PVE (%)**	**Additive effect**	**Flanking markers**		**Closest marker**
								**Left marker**	**Right marker**	
1	ICCV 96029 × CDC Frontier	*Qefl1*-1	3	0.00	3.45	5.66	−4.40	CaM1122	TR13	CaM1122
		*Qefl1*-2	4	41.00	5.66	11.75	−5.27	GAA47	ICCM0192a	GAA47
2	ICC 5810 × CDC Frontier	*Qefl2*-1	1	15.00	12.88	20.28	−3.23	TA122	TA30	TA30
		*Qefl2*-2	3	21.00	16.70	24.95	−6.19	CaM1358	TA142	TA142
		*Qefl2*-3	4	55.00	9.18	10.53	−4.41	NCPGR21	GAA47	GAA47
		*Qefl2*-4	8	15.00	17.79	25.73	−7.05	GA6	TA118	TA118
3	BGD 132 × CDC Frontier	*Qefl3*-1	3	5.00	5.24	4.39	−1.23	CaM1515	TR13	TR13
		*Qefl3*-2	3	31.00	4.21	4.04	−3.36	TA142	TA64	TA142
		*Qefl3*-3	8	2.00	44.38	64.95	−13.0	TA127	H1D24	H1D24
4	ICC 16641 × CDC Frontier	*Qefl4*-1	6	9.00	55.60	88.19	−16.74	TA14	TR44	TR44

#### ICC 5810 × CDC frontier

Four major QTLs were identified for flowering time in this cross (Table 3, Supplementary Table 6, Supplementary Figures 2, 6). The QTL “*Qefl2*-1” (LOD = 12.88; PVE = 20.28%) was identified on CaLG01 flanked by the markers TA122 and TA30. Another QTL “*Qefl2*-2” (LOD = 16.70; PVE = 24.95%) was located on CaLG03 in the marker interval CaM1358-TA142. Third QTL “*Qefl2*-3” was detected on CaLG04 (LOD = 9.18; PVE = 10.53%) between the markers NCPGR21 and GAA47. Similarly, the QTL “*Qefl2*-4” was identified between the markers GA6 and TA118 on CaLG08 (LOD = 17.79) which accounted for 25.73% of PVE. The estimated additive effect was negative for all the QTLs suggesting that the allele for early flowering at this loci was contributed by ICC 5810.

#### BGD 132 × CDC frontier

One major and two minor QTLs were detected for the flowering time (Table 3, Supplementary Table 6 and Supplementary Figures 3, 7). The major QTL “*Qefl3*-3” was located on CaLG08 in the marker interval TA127-H1D24. This was a highly significant QTL with a LOD peak value of 44.38 and PVE of 64.95%. The minor QTLs *Qefl3*-1 (LOD = 5.24; PVE = 4.39%) and *Qefl3*-2 (LOD = 4.21; PVE = 4.04%) were also detected on CaLG03 defined by marker intervals CaM1515-TR13 and TA142-TA64, respectively. In this cross also all the QTLs showed negative additive effect indicating that allele for early flowering at this locus was contributed by BGD 132.

#### ICC 16641 × CDC frontier

A single major QTL (*Qefl4*-1) for flowering time was identified on CaLG06 flanked by markers TA14 and TR44 (Table 3, **Figure 4B**, Supplementary Table 6, and Supplementary Figure 4). This QTL contributed a high PVE of 88.19% at LOD value of 55.60. This QTL also showed a negative additive effect suggesting that allele for early flowering at this locus was contributed by ICC 16641. In this study, if more than one QTL of different cross share one or two flanking markers in common, it was considered as only one genomic region. The sequences of the markers flanking the QTL regions are provided (Supplementary Table 5).

## Discussion

Flowering time is an important trait for adaption of chickpea particularly in the semi-arid environments (Kumar and Abbo, 2001; Gaur et al., 2015). Information on genetic and molecular basis of flowering behavior would be useful for the breeding programs focusing on development of early maturing varieties. So far, four genes (*efl-1, ppd/efl-2, efl-3* and *efl-4*) controlling flowering time in ICCV 96029 (Kumar and van Rheenen, 2000), ICC 5810 (Or et al., 1999; Hegde, 2010), BGD 132 (Hegde, 2010) and ICC 16641 (Gaur et al., 2015) have been reported in chickpea. When these early flowering lines were crossed with a late flowering cultivar (CDC Frontier), F_1_s were late to flower as the gene for delayed flowering is known to be dominant to early flowering in chickpea (Or et al., 1999; Kumar and van Rheenen, 2000; Hegde, 2010; Gaur et al., 2015). Segregation analysis (in F_2_ and F_3_) revealed monogenic inheritance of flowering time in the crosses ICCV 96029 × CDC Frontier, BGD 132 × CDC Frontier and ICC 16641 × CDC Frontier. Whereas, in ICC 5810 × CDC Frontier, it was under digenic control with complementary effect. Therefore, the present study confirmed the single recessive gene hypothesis for flowering time in ICCV 96029 (Kumar and van Rheenen, 2000), BGD 132 (Hegde, 2010) and ICC 16641 (Gaur et al., 2015) and the digenic control in ICC 5810 (Hegde, 2010; Gaur et al., 2015). This implies that the early flowering trait can be easily incorporated into the desired genetic backgrounds.

In the present study though sufficient number of SSRs (472) that represent most of the chickpea genome were used, a low polymorphism level (21.40, 20.13, 19.07 and 19.70%) was observed compared to previous studies (Tar'an et al., 2007; Kottapalli et al., 2009). The goodness of fit of the observed segregation ratio to the expected ratio demonstrated that the majority of the SSRs did not significantly deviate from the expected 1:2:1 ratio (*P* ≥ 0.05). Since F_2_ populations were used, the intraspecific maps developed in this study represent the coarse genetic maps spanning 248.58, 331.37, 311.10 and 385.13 cM in ICCV 96029 × CDC Frontier, ICC 5810 × CDC Frontier, BGD 132 × CDC Frontier and ICC 16641 × CDC Frontier, respectively. These results indicate that the intraspecific maps obtained are less dense compared to earlier maps (Winter et al., 2000; Nayak et al., 2010; Thudi et al., 2011; Varshney et al., 2014). Also, a varying levels of marker distributions were observed with dense sub-clusters of marker loci either in the central region or at distal ends of most of the linkage groups in all the maps. It may reflect the low level of recombination in centromeric and subtelomeric genomic regions (Tanksley et al., 1992) and such apparent clustering of markers on the linkage groups was also observed in the previous studies (Tanksley et al., 1992; Winter et al., 1999; Millan et al., 2010; Nayak et al., 2010). When these genetic maps were compared with earlier maps, the SSRs that are common between the current maps and the previous maps (Millan et al., 2010; Thudi et al., 2011; Jaganathan et al., 2014; Varshney et al., 2014) were placed on the same linkage groups which encourages the possibility of integration of different maps through common markers. However, the order of marker loci on intra-specific maps differed in several instances.

Construction of a consensus map based on synteny between the several linkage maps will make it possible to expand the chickpea genetic map and increase the marker density. In the present study, the data sets from four populations were joined to develop the consensus map. While comparing four intra-specific genetic maps, 28 loci were found common between four maps. These were considered as anchor markers and used for merging the genetic maps for construction of the consensus genetic map. The consensus map developed contained 109 markers that covered 363.85 cM of map length, which is less dense compared to the consensus maps of Millan et al. (2010) and Varshney et al. (2014). A detailed comparison between individual component maps and consensus map reflects a general coincidence. Although differences in marker order exist, linkage groups are generally conserved. The sub clusters and gaps were also observed in most of the LGs either at central or in distal regions. Since the consensus map is low in marker density, saturating these regions with more markers will help in any map based cloning of agronomically important genes.

In the present study, 10 genomic regions were identified for flowering time including seven QTLs having major effects with PVE more than 10%. A major QTL “*Qefl1*-2” (PVE = 11.75%) for flowering time was detected in ICCV 96029 × CDC Frontier on CaLG04 (flanked by GAA47-ICCM192a). Mendelian inheritance revealed that flowering time was governed by a single major gene. The identified QTL on CaLG04 could be same as the chromosomal region reported by Daba et al. (2016) who mapped four QTLs for days to flowering on LG4 in the same cross. Previously, Cobos et al. (2007) reported a major QTL for days to 50% flowering (QTL_*DF*1_; 20% PVE) on LG4. This QTL had a common marker (i.e., GAA47) with the QTL reported in this study. Therefore, they may refer to the same QTL. In the present study, a minor QTL “*Qefl1*-1” (PVE = 6.90%) was also identified on CaLG03. Recently, Daba et al. (2016) also mapped a minor QTL on LG3 in the same cross. Therefore, these QTLs may be representing the same genomic region. Cho et al. (2002) and Jamalabadi et al. (2013) also reported a QTL for days to flowering on LG3 using a RIL population from a cross involving ICCV 2 as one of the parents. The line ICCV 2 is an indirect source of earliness (*efl-1*) in our study. However, lack of common markers does not allow a definitive conclusion that these two QTLs represent the same locus. Daba et al. (2016) identified additional major QTLs for days to flowering on LG5 and LG8 that are consistent across years and sites. However, no such QTLs were identified in the present study since we evaluated the mapping population at only one location. Based on these findings it is apparent that several unknown factors confer time to flowering in chickpea even though segregation for a major flowering gene was observed. Similar findings were also reported earlier (Cho et al., 2002).

In ICC 5810 × CDC Frontier, major QTLs on CaLG01 (*Qefl2*-1, PVE = 20.28%), CaLG03 (*Qefl2*-2, PVE = 24.95%), CaLG04 (*Qefl2*-3, PVE = 10.53%) and CaLG08 (*Qefl2*-4, PVE = 25.73%) were identified. Genetic studies revealed that two major genes with complementary gene action controlling flowering time in this cross. Earlier, Cho et al. (2002) detected a single QTL for flowering time on LG3 between the markers TS57 and TA127. Recently, Jamalabadi et al. (2013) also identified a QTL for flowering time on LG3 closely linked to the marker TA117. However, these markers were not mapped in the present study and hence the exact chromosomal location could not be compared. Whereas, Cobos et al. (2009) and Aryamanesh et al. (2010) mapped a QTL for flowering time on LG3 closely linked to marker TA142 which was also detected in this study. Therefore, these QTLs could belong to the same set of genes. Another QTL for flowering time was identified by Cobos et al. (2007) on LG4 (explaining 20% PVE) closely linked to the marker GAA47. A QTL on CaLG4 (*Qefl2*-3, PVE = 10.53%) was detected having GAA47 as flanking marker in the present study. Therefore, these QTLs could be same in both the findings. In all these studies, different parental lines were used. However, Lichtenzveig et al. (2006) in the cross involving Hadas and ICC 5810 reported three QTLs on LG1, LG2 and LG8 for flowering time. Recently, Rehman et al. (2011) reported four QTLs for flowering time on LG1, LG3, LG4 and LG8. None of these QTLs were found similar to the QTLs detected in the present study. In our study, however, LG2 was not associated with any effect on flowering time. One possible explanation for this could be the absence of common markers in our map due to non-availability of more number of polymorphic markers for linkage analysis. Further studies are needed to confirm which two major genes out of four QTLs detected in this study are responsible for flowering time in ICC 5810.

**Figure 3:**
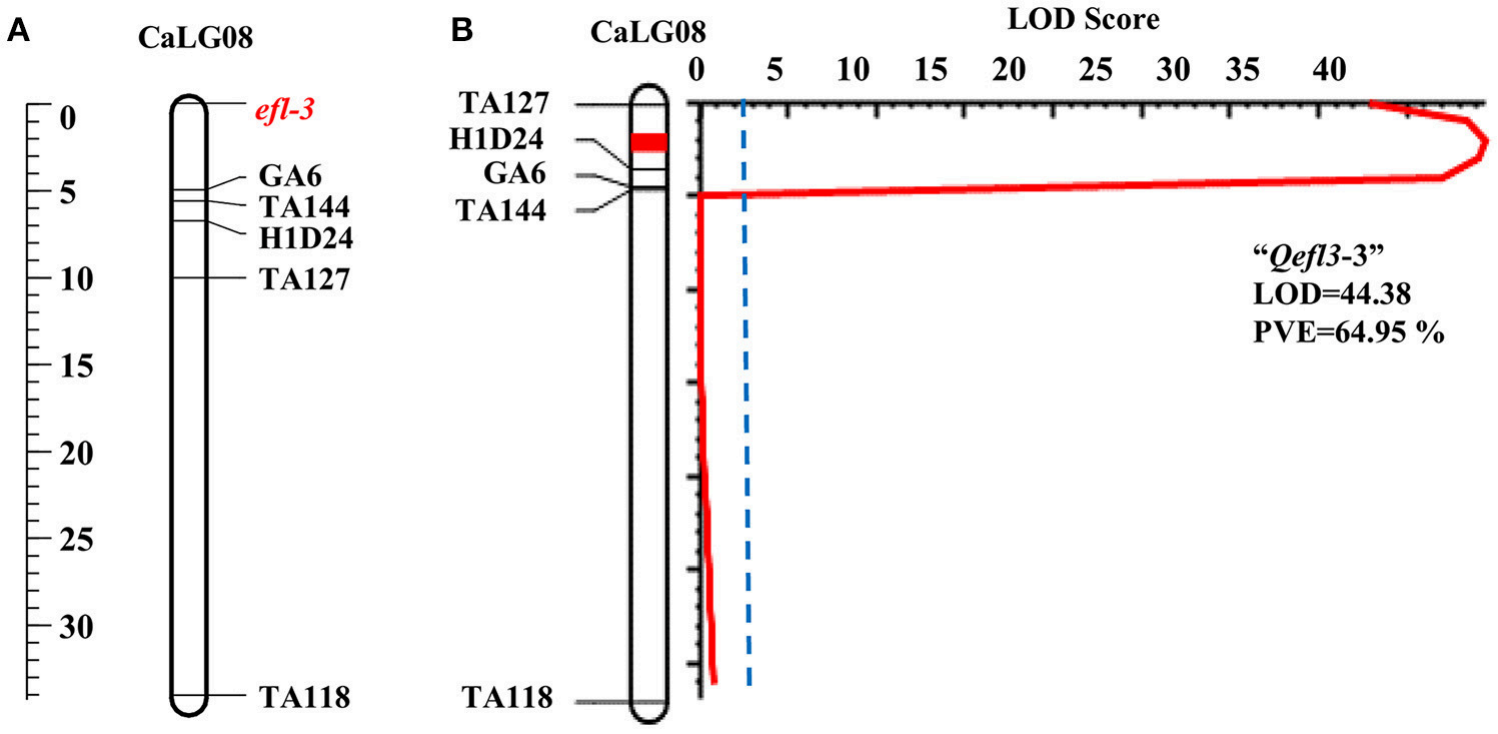
Mapping of major flowering time gene “*efl-3*” on CaLG08 of the cross BGD 132 × CDC Frontier. **(A)** Mapping of major flowering time gene “*efl-3*” on CaLG08 based on F_3_ segregating data **(B)** Mapping of major QTL for flowering time “*Qefl3*-3” on CaLG08.

**Figure 4:**
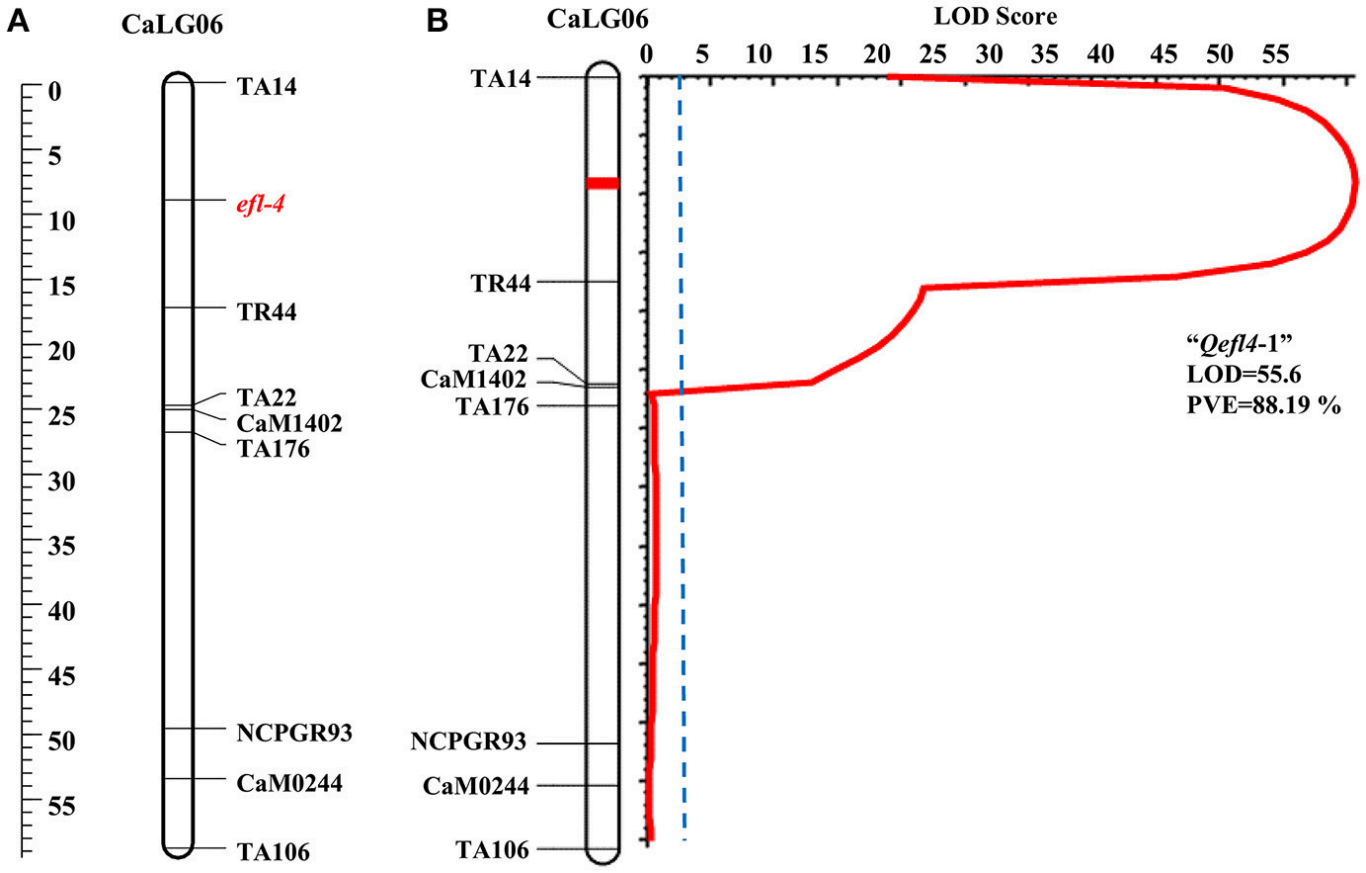
Mapping of major flowering time gene “*efl-4*” on CaLG06 of the cross ICC 16641 × CDC Frontier. **(A)** Mapping of major flowering time gene “*efl-4*” on CaLG06 based on F_3_ segregating data **(B)** Mapping of major QTL for flowering time “*Qefl4*-1” on CaLG06.

In BGD 132 × CDC Frontier, a major QTL “*Qefl3*-3” (flanked by markers TA127 and H1D24) was detected for flowering time on CaLG08 with 64.95% PVE (Figure 3B). This is the first report of mapping major flowering time gene “*efl-3*” in BGD 132. Linkage analysis based on F_3_ segregating data resulted in mapping of flowering time locus “*efl-3*” on CaLG08 in this cross (Figure 3A). Previously, Cho et al. (2002) reported a QTL for flowering time on LG3 flanked by markers TS57 and TA127. However, LG3 of Cho et al. (2002) is equivalent to CaLG8 in this study based on the common markers of the current map and genetic maps of Tar'an et al. (2007) and Varshney et al. (2014). It appears that both the QTLs could be the same. Two additional minor QTLs i.e., “*Qefl3*-1” and “*Qefl3*-2” were also detected on CaLG03. Hence, CaLG08 appears to be a strong candidate linkage group having major QTL controlling flowering time gene “*efl-3*.” In ICC 16641 × CDC Frontier, a single putative QTL (*Qefl4*-1; PVE = 88.19%) for flowering time was detected on CaLG06 between markers TA14 and TR44 (Figure 4B). This novel QTL is unique for the flowering time gene “*efl-4*” and is reported for the first time in this study. Mendelian inheritance also revealed monogenic inheritance of flowering time in this cross. This was further confirmed by linkage analysis and mapping of major flowering time gene “*efl-4*” on CaLG06 of the chickpea genetic map between the markers TA14 and TR44 (Figure 4A). Therefore, this genomic region can be targeted for developing early maturing chickpea varieties through Marker Assisted Breeding (MAB).

## Conclusions

The present study revealed major gene inheritance of flowering time genes under short season environment typical to semi-arid tropics. This simple inheritance of early flowering trait can be easily transferred into desired genetic backgrounds. The SSRs were used to construct separate intraspecific linkage maps of four F_2_ populations. These linkage maps were combined to construct a consensus map of chickpea with 109 marker loci (363.85 cM). QTL analysis revealed altogether 10 genomic regions for flowering time including seven major QTLs distributed across CaLG01, CaLG03, CaLG04, CaLG06 and CaLG08 of chickpea genetic map. It is also the first report on mapping of flowering time genes “*efl-3*” and “*efl-4*” in chickpea. These genomic regions provide strong basis for further investigation on fine mapping and validation of the identified QTLs which will help in developing early-maturing chickpea varieties under short season environments.

## Author contributions

PG coordinated this project. PG, KV, RV, SS, MT, SBS and AP guided BM in planning and designing this study. BM, PG, SS and SBS were involved in developing mapping populations, designing field experiments, phenotyping and data analysis. BM, RV, MT and AK were involved in DNA isolation, genotyping, linkage maps construction and QTL analysis. BM, PG, SS, KV, MT and AP drafted the manuscript and all the authors reviewed and approved the final version of the manuscript.

### Conflict of interest statement

The authors declare that the research was conducted in the absence of any commercial or financial relationships that could be construed as a potential conflict of interest.

## Supplementary material

Supplementary Figure 1 Genetic linkage map of the cross ICCV 96029 × CDC Frontier with 75 loci spanning 248.76 cM. The genetic distance in cM is represented on left hand side and the markers names are on the right hand side of the linkage group. The QTLs identified for flowering time in this cross are shown here.

Supplementary Figure 2 Genetic linkage map of the cross ICC 5810 × CDC Frontier with 75 loci spanning 331.37 cM. The genetic distance in cM is represented on left hand side and the markers names are on the right hand side of the linkage group. The QTLs identified for flowering time in this cross are shown here.

Supplementary Figure 3 Genetic linkage map of the cross BGD 132 × CDC Frontier with 68 loci spanning 311.10 cM. The genetic distance in cM is represented on left hand side and the markers names are on the right hand side of the linkage group. The QTLs identified for flowering time in this cross are shown here.

Supplementary Figure 4 Genetic linkage map of the cross ICC 16641 × CDC Frontier with 67 loci spanning 385.13 cM. The genetic distance in cM is represented on left hand side and the markers names are on the right hand side of the linkage group. The QTLs identified for flowering time in this cross are shown here.

Supplementary Figure 5 QTLs identified for flowering time in the cross ICCV 96029 × CDC Frontier. A major QTL on CaLG04 and a minor QTL on CaLG03 were identified for flowering time in this cross.

Supplementary Figure 6 QTLs identified for flowering time in the cross ICC 5810 × CDC Frontier. A major QTL each on CaLG01, CaLG03, CaLG04, and CaLG08 were identified for flowering time in this cross.

Supplementary Figure 7 QTLs identified for flowering time in the cross BGD 132 × CDC Frontier. A major QTL on CaLG08 and two minor QTLs on CaLG03 were identified for flowering time in this cross.

Supplementary Table 1 Mean and range of variation of flowering time and maturity in parental lines.

Supplementary Table 2 Mean and range of variation of flowering time and maturity in F_1_s and F_2_ populations.

Supplementary Table 3 Segregation of flowering time in F_3_ progenies of four chickpea crosses.

Supplementary Table 4 Polymorphism status of SSR markers between parental lines of four intraspecific mapping populations.

Supplementary Table 5 Primer sequences of the markers flanking flowering time QTL regions in four chickpea crosses.

Supplementary Table 6 Coordinates for the flowering time QTLs identified in four chickpea crosses.

## References

[B1] Anbessa Y. Warkentin T. Vandenberg A. Ball R. (2006). Inheritance of time to flowering in chickpea in a short-season temperate environments. J. Hered. 97, 55–61. 10.1093/jhered/esj009 16394253

[B2] Aryamanesh N. Nelson M. N. Yan G. Clarke H. J. Siddique K. H. M. (2010). Mapping a major gene for growth habit, QTLs for ascochyta blight resistance, flowering time in a population between chickpea, Cicer reticulatum. Euphytica 173, 307–319. 10.1007/s10681-009-0086-2

[B3] Bohra A. Saxena R. K. Gnanesh B. N. Saxena K. B. Byregowda M. Rathore. (2012). An intra-specific consensus genetic map of pigeonpea (*Cajanus cajan* (L) Millspaugh) derived from six mapping populations. Theor. Appl. Genet. 125, 1325–1338. 10.1007/s00122-012-1916-5 22772726 PMC3442162

[B4] Cho S. Kumar J. Shultz J. L. Anupama K. Tefera F. Muehlbauer F. J. (2002). Mapping genes for double podding, other morphological traits in chickpea. Euphytica 128, 285–292. 10.1023/A:1020872009306

[B5] Churchill G. A. Doerge R. W. (1994). Empirical threshold values for quantitative trait mapping. Genetics 138, 963–971. 7851788 10.1093/genetics/138.3.963 PMC1206241

[B6] Cobos M. J. Rubio J. Romero F. M. D. Garza R. Moreno M. T. Millan T. (2007). Genetic analysis of seed size, yield, days to flowering in a chickpea recombinant inbred line population derived from a Kabuli x Desi cross. Ann. Appl. Biol. 151, 33–42. 10.1111/j.1744-7348.2007.00152.x

[B7] Cobos M. J. Winter P. Kharrat M. Cubero J. I. Gil J. Millan T. (2009). Genetic analysis of agronomic traits in a wide cross of chickpea. Field Crop Res. 111, 130–136. 10.1016/j.fcr.2008.11.006

[B8] Cuc L. M. Mace E. Crouch J. Quang V. D. Long T. D. Varshney R. K. (2008). Isolation, characterization of novel microsatellite markers, their application for diversity assessment in cultivated groundnut (*Arachis hypogaea*). BMC Plant Biol. 8:55. 10.1186/1471-2229-8-55 18482440 PMC2416452

[B9] Daba K. Deokar A. Banniza S. Warkentin T. D. Tar'an T. (2016). QTL mapping of early flowering and resistance to ascochyta blight in chickpea. Genome 59, 413–425. 10.1139/gen-2016-0036 27244453

[B10] FAOSTAT (2015). Available online at: http://faostat3.fao.org/download/Q/QC/E (Accessed: May 10, 2015)

[B11] Gaur P. M. Jukanti A. K. Srinivasan S. Chaturvedi S. K. Basu P. S. Babbar A. (2014). Climate change, heat stress tolerance in chickpea, in Climate Change, Plant Abiotic Stress Tolerance, ed Tuteja N. Gill S. S. (Weinheim: Wiley-VCH Verlag GmbH Co, KGaA), 839–855.

[B12] Gaur P. M. Kumar J. Gowda C. L. L. Pande S. Siddique K. H. M. Khan T. N. (2008). Breeding chickpea for early phenology: perspectives, progress, prospects, in Food Legumes for Nutritional Security, Sustainable Agriculture, Vol. 2, ed Kharkwal M. C. (New Delhi: Indian Society of Genetics, Plant Breeding), 39–48.

[B13] Gaur P. M. Samineni S. Tripathi S. Varshney R. K. Gowda C. L. L. (2015). Allelic relationships of flowering time genes in chickpea. Euphytica 203, 295–308. 10.1007/s10681-014-1261-7

[B14] Gaur R. Sethy N. K. Choudhary S. Shokeen B. Gupta V. Bhatia S. (2011). Advancing the STMS genomic resources for defining new locations on the intra-specific genetic linkage map of chickpea (*Cicer arietinum* L.). BMC Genomics 12:117 10.1186/1471-2164-12-117 21329497 PMC3050819

[B15] Gumber R. K. Sarvjeet S. (1996). Genetics of flowering time in chickpea: a preliminary report. Crop Improv. 23, 295–296.

[B16] Hegde V. S. (2010). Genetics of flowering time in chickpea in a semi-arid environment. Plant Breed. 129, 683–687. 10.1111/j.1439-0523.2009.01748.x

[B17] Hossain S. Ford R. McNeil D. L. Pittock C. Pannozzo J. F. (2010). Development of a selection tool for seed shape, QTL analysis of seed shape with other morphological traits for selective breeding in chickpea (*Cicer arietinum* L.). Aust. J. Crop Sci. 4, 278–288.

[B18] Jaganathan D. Thudi M. Kale M. Azam S. Roorkiwal M. Gaur P. M.. (2014). Genotyping-by-sequencing based intra-specific genetic map refines a “QTL-hotspot” region for drought tolerance in chickpea. Mol. Genet. Geno. 290, 559–571. 10.1007/s00438-014-0932-3 25344290 PMC4361754

[B19] Jamalabadi J. G. Saidi A. Karami E. Kharkesh M. Talebi R. (2013). Molecular mapping, characterization of genes governing time to flowering, seed weight, plant height in an intraspecific genetic linkage map of chickpea (*Cicer arietinum*). Biochem Genet. 51, 387–397. 10.1007/s10528-013-9571-3 23371372

[B20] Karami E. Talebi R. Kharkesh M. Saidi A. (2015). A linkage map of chickpea (*Cicer arietinum* L.) based on population from ILC 3279 × ILC 588 crosses: location of genes for time to flowering, seed size and plant height. Genetika 47, 253–263. 10.2298/GENSR1501253K

[B21] Kottapalli P. Gaur P. M. Katiyar S. K. Crouch J. H. Buhariwalla H. K. Pande S. (2009). Mapping, validation of QTLs for resistance to an Indian isolate of Ascochyta blight pathogen in chickpea. Euphytica 165, 79–88. 10.1007/s10681-008-9762-x

[B22] Kumar J. Abbo S. (2001). Genetics of flowering time in chickpea, its bearing on productivity in semi-arid environments. Adv. Agron. 72, 107–138. 10.1016/S0065-2113(01)72012-3

[B23] Kumar J. van Rheenen H. A. (2000). A major gene for time of flowering in chickpea. J. Hered. 91, 67–68. 10.1093/jhered/91.1.67 10739130

[B24] Lichtenzveig J. Bonfil D. J. Zhang H. B. Shtienberg D. Abbo S. (2006). Mapping quantitative trait loci in chickpea associated with time to flowering, resistance to *Didymella rabiei*, the causal agent of Ascochyta blight. Theor. Appl. Genet. 113, 1357–1369. 10.1007/s00122-006-0390-3 17016689

[B25] Lichtenzveig J. Scheuring C. Dodge J. Abbo S. Zhang H. B. (2005). Construction of BAC, BIBAC libraries, their applications for generation of SSR markers for genome analysis of chickpea, *Cicer arietinum* L. Theor. Appl. Genet. 110, 492–510. 10.1007/s00122-004-1857-8 15712010

[B26] Microsoft Excel (2013). Microsoft Corporation 1985. Redmond. Available online at: www.microsoft.com

[B27] Millan T. Winter P. Jüngling R. Gil J. Rubio J. Cho S. (2010). A consensus genetic map of chickpea (*Cicer arietinum* L.) based on 10 mapping populations. Euphytica 175, 175–189. 10.1007/s10681-010-0157-4

[B28] Nayak S. N. Zhu H. Varghese N. Choi H. K. Datta S. Horres R.. (2010). Integration of novel SSR, gene-based marker loci in the chickpea genetic map, establishment of new anchor points with *Medicago truncatula* genome. Theor. Appl. Genet. 120, 1415–1441. 10.1007/s00122-010-1265-1 20098978 PMC2854349

[B29] Or E. Hovav R. Abbo S. (1999). A major gene for flowering time in chickpea. Crop Sci. 39, 315–322.

[B30] Rehman A. U. Malhotra R. S. Bett K. Tar'an B. Bueckert R. Warkentin T. D. (2011). Mapping QTL associated with traits affecting grain yield in chickpea (*Cicer arietinum* L.) under terminal drought stress. Crop Sci. 51, 450–463. 10.2135/cropsci2010.03.0129

[B31] Sethy N. K. Shokeen B. Edwards K. J. Bhatia S. (2006). Development of microsatellite markers, analysis of intraspecific genetic variability in chickpea (*Cicer arietinum* L.). Theor. Appl. Genet. 112, 1416–1428. 10.1007/s00122-006-0243-0 16534564

[B32] Subbarao G. V. Johansen C. Slinkard A. E. Rao R. C. N. Saxena N. P. Chauhan Y. S. (1995). Strategies for improving drought resistance in grain legumes. CRC Crit. Rev. Plant Sci. 14, 469–523. 10.1080/07352689509701933

[B33] Tanksley S. D. Ganal M. W. Prince J. P. de Vicente M. C. Bonierbale M. W. Broun P. (1992). High density molecular map linkage maps of the tomato, potato genomes. Genetics 132, 1141–1160. 1360934 10.1093/genetics/132.4.1141 PMC1205235

[B34] Tar'an B. Warkentin T. D. Tullu A. Vandenberg A. (2007). Genetic mapping of Ascochyta blight resistance in chickpea (*Cicer arietinum* L.) using a simple sequence repeat linkage map. Genome 50, 26–34. 10.1139/g06-137 17546068

[B35] Than A. M. Maw J. B. Aung T. Gaur P. M. Gowda C. L. L. (2007). Development, adoption of improved chickpea varieties in Myanmar. J. SAT. Agric. Res. 5, 1–3.

[B36] Thudi M. Bohra A. Nayak S. N. Varghese N. Shah T. M. Penmetsa R. V.. (2011). Novel SSR markers from BAC-end sequences, DArT arrays, a comprehensive genetic map with 1,291 marker loci for chickpea (*Cicer arietinum* L.). PLoS ONE 6:e272275. 10.1371/journal.pone.0027275 22102885 PMC3216927

[B37] Upadhyaya H. D. Bajaj D. Das S. Saxena M. S. Badoni S. Kumar V.. (2015). A genome-scale integrated approach aids in genetic dissection of complex flowering time trait in chickpea. Plant Mol. Biol. 89, 403–420. 10.1007/s11103-015-0377-z 26394865

[B38] Van Ooijen J. W. (2006). JoinMap ® 4: Software for the Calculation of Genetic Linkage Maps in Experimental Populations. Wageningen: Kyazma, BV.

[B39] Varshney R. K. Song C. Saxena R. K. Azam S. Yu S. Sharpe A. G.. (2013). Draft genome sequence of chickpea (*Cicer arietinum*) provides a resource for trait improvement. Nat. Biotechnol. 31, 240–246. 10.1038/nbt.2491 23354103

[B40] Varshney R. K. Thudi M. Nayak S. N. Gaur P. M. Kashiwagi J. Krishnamutrhy L.. (2014). Genetic dissection of drought tolerance in chickpea (*Cicer arietinum* L.). Theor. Appl. Genet. 127, 445–462. 10.1007/s00122-013-2230-6 24326458 PMC3910274

[B41] Wang J. Li H. L. Zhang L. Meng L. (2014). Users' Manual of QTL IciMapping. The Quantitative Genetics Group, Institute of Crop Science, Chinese Academy of Agricultural Sciences (CAAS). Beijing; Mexico: Genetic Resources Program, International Maize, Wheat Improvement Center (CIMMYT), Apdo.

[B42] Warkentin T. Banniza S. Vandenberg A. (2005). CDC Frontier kabuli chickpea. Can. J. Plant Sci. 85, 909–910. 10.4141/P04-185

[B43] Warkentin T. Vandenberg A. Banniza S. Tar'an B. Tullu A. Lulsdorf M. (2003). Breeding chickpea for improved Ascochyta blight resistance, early maturity in western Canada, in Proceedings of International Chickpea Conference, Indira Gandhi Agricultural University, 20-22 Jan, eds Sharma R. N. Yasin M. Swami S. L. Khan M. A. William A. J. (Raipur), 1–4.

[B44] Winter P. Benko-Isepp A. M. Huttel B. Ratnaparkhe M. Tullu A. Sonnante G. (2000). A linkage map of the chickpea (*Cicer arietinum* L.) genome based on recombinant inbred lines from a *C. arietinum* x *C. reticulatum* cross; localization of resistance genes for fusarium wilt races 4, 5. Theor. Appl. Genet. 101, 1155–1163. 10.1007/s001220051592

[B45] Winter P. Pfaff T. Udupa S. M. Hüttel B. Sharma P. C. Sahi S.. (1999). Characterization, mapping of sequence-tagged microsatellite sites in the chickpea (C. arietinum L.). Mol. Gen. Genet. 262, 90–101. 10.1007/s004380051063 10503540

