# Supplementary figures and images for: Molecular Mapping of Flowering Time Major Genes and QTLs in Chickpea (Cicer arietinum L.)
Source: Front Plant Sci. 2017 Jul 6;8:1140. doi: 10.3389/fpls.2017.01140 (PMC5498527; doi:10.3389/fpls.2017.01140)

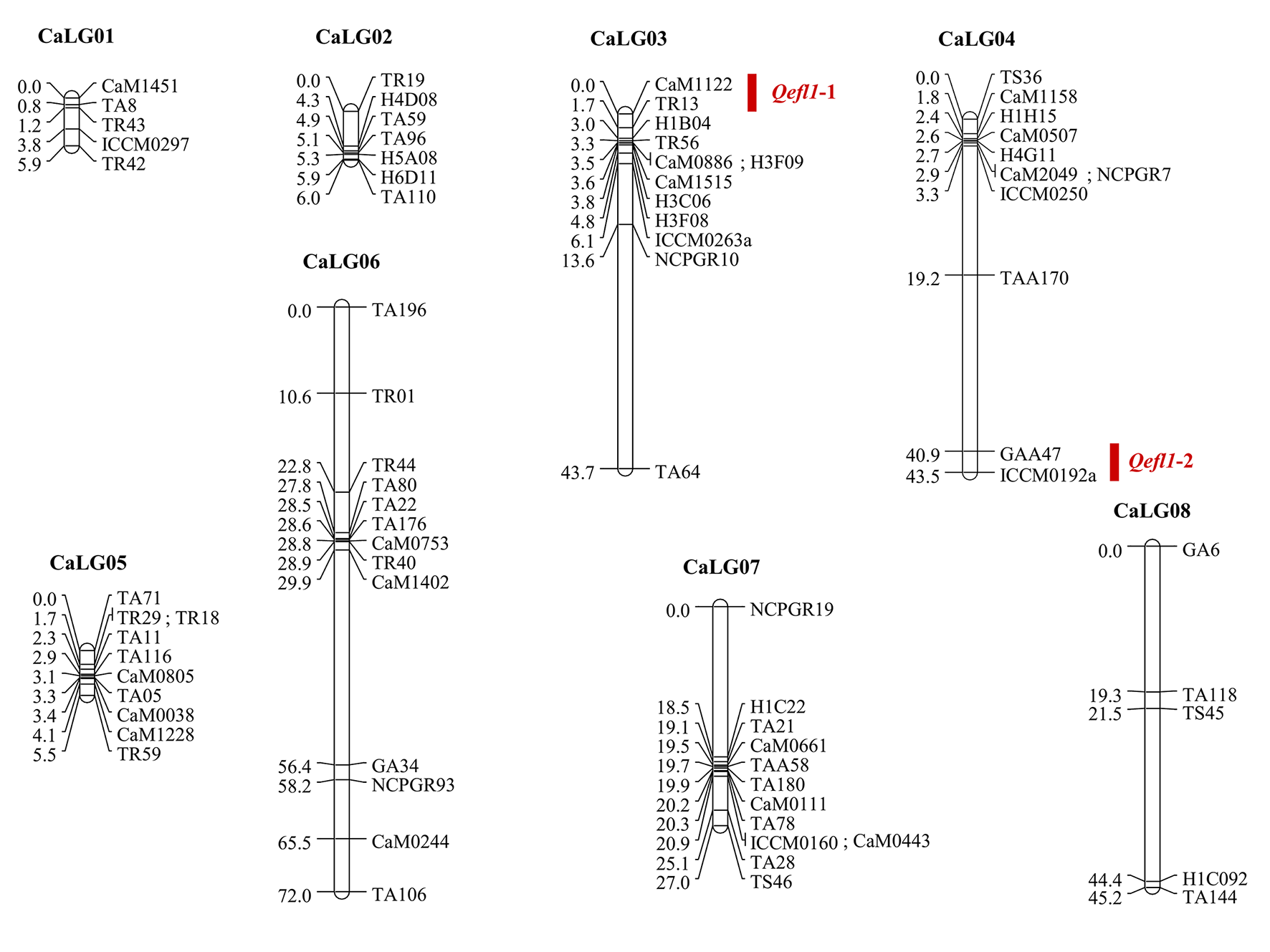

Supplement: Supplementary Figure 1 — Genetic linkage map of the cross ICCV 96029 × CDC Frontier with 75 loci spanning 248.76 cM. The genetic distance in cM is represented on left hand side and the markers names are on the right hand side of the linkage group. The QTLs identified for flowering time in this cross are shown here. [file Image1.TIF]

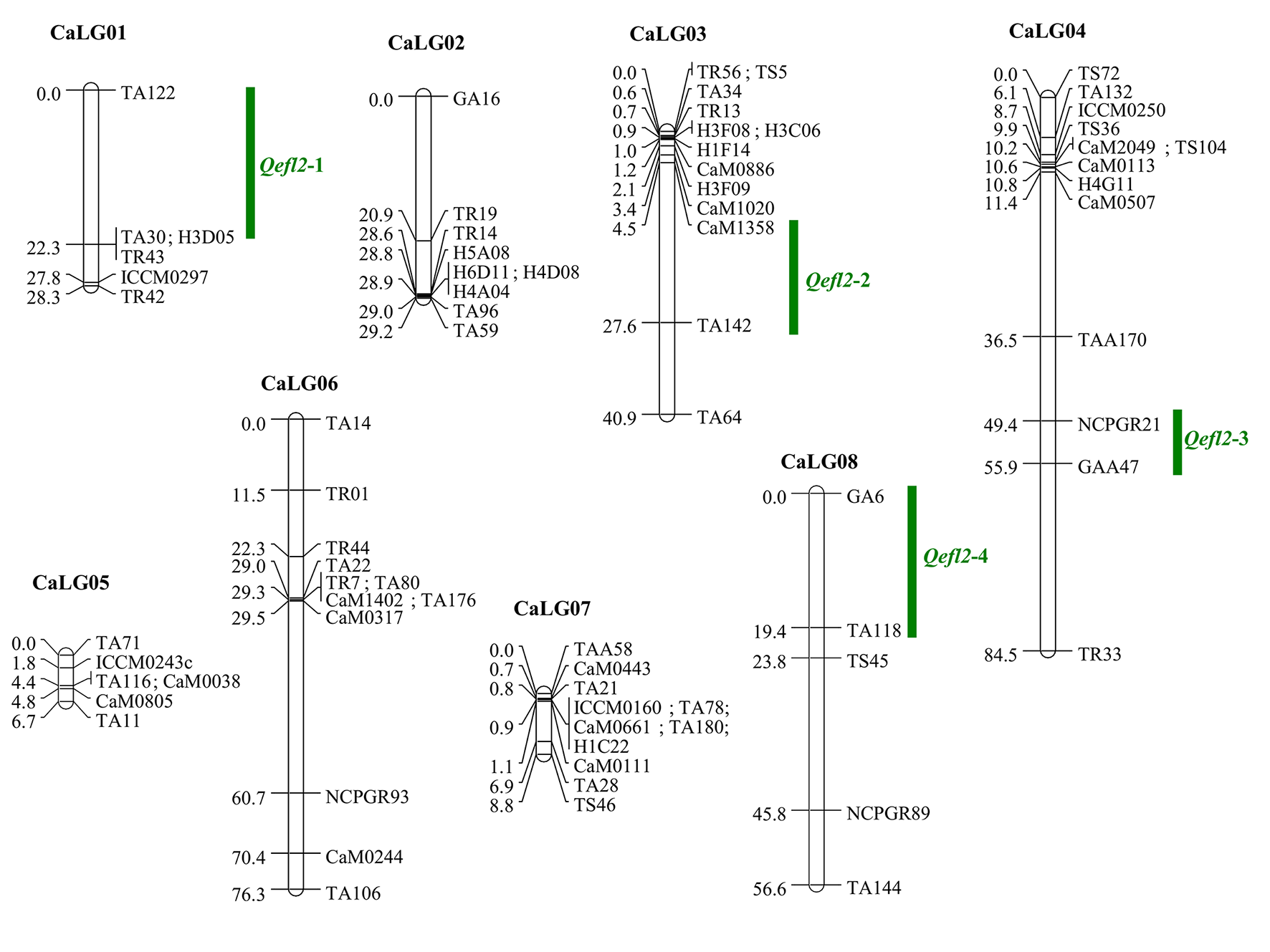

Supplement: Supplementary Figure 2 — Genetic linkage map of the cross ICC 5810 × CDC Frontier with 75 loci spanning 331.37 cM. The genetic distance in cM is represented on left hand side and the markers names are on the right hand side of the linkage group. The QTLs identified for flowering time in this cross are shown here. [file Image2.TIF]

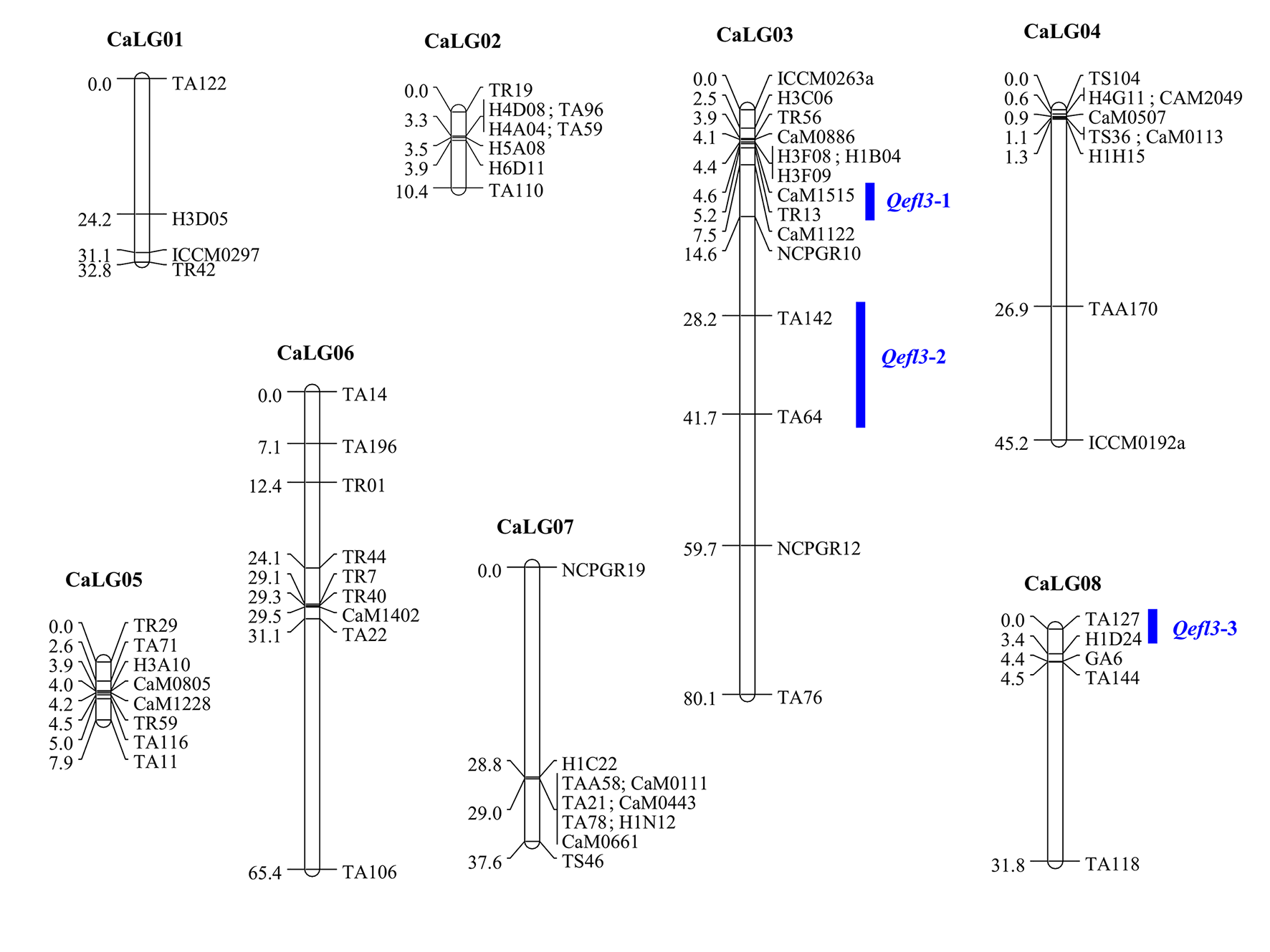

Supplement: Supplementary Figure 3 — Genetic linkage map of the cross BGD 132 × CDC Frontier with 68 loci spanning 311.10 cM. The genetic distance in cM is represented on left hand side and the markers names are on the right hand side of the linkage group. The QTLs identified for flowering time in this cross are shown here. [file Image3.TIF]

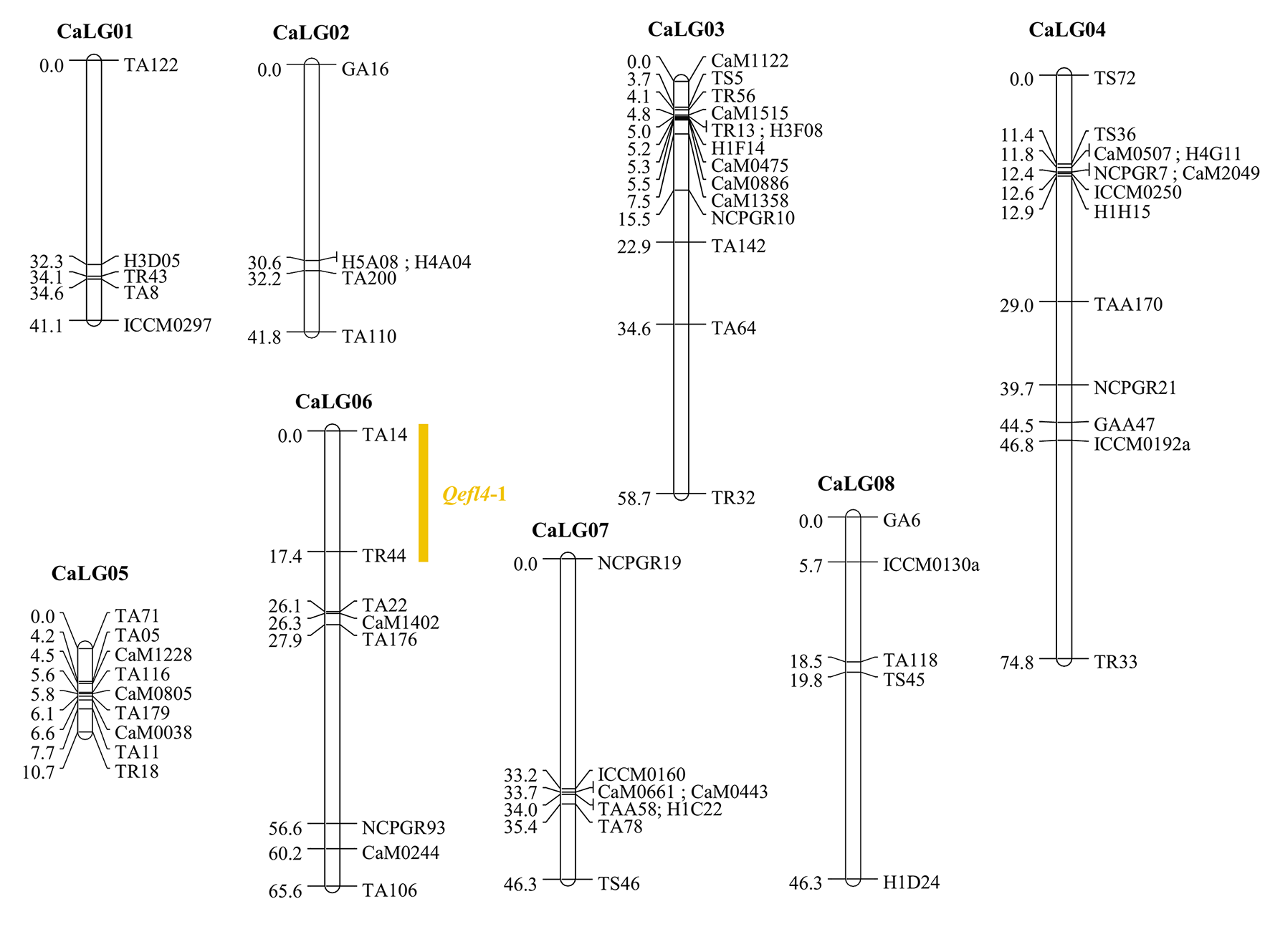

Supplement: Supplementary Figure 4 — Genetic linkage map of the cross ICC 16641 × CDC Frontier with 67 loci spanning 385.13 cM. The genetic distance in cM is represented on left hand side and the markers names are on the right hand side of the linkage group. The QTLs identified for flowering time in this cross are shown here. [file Image4.TIF]

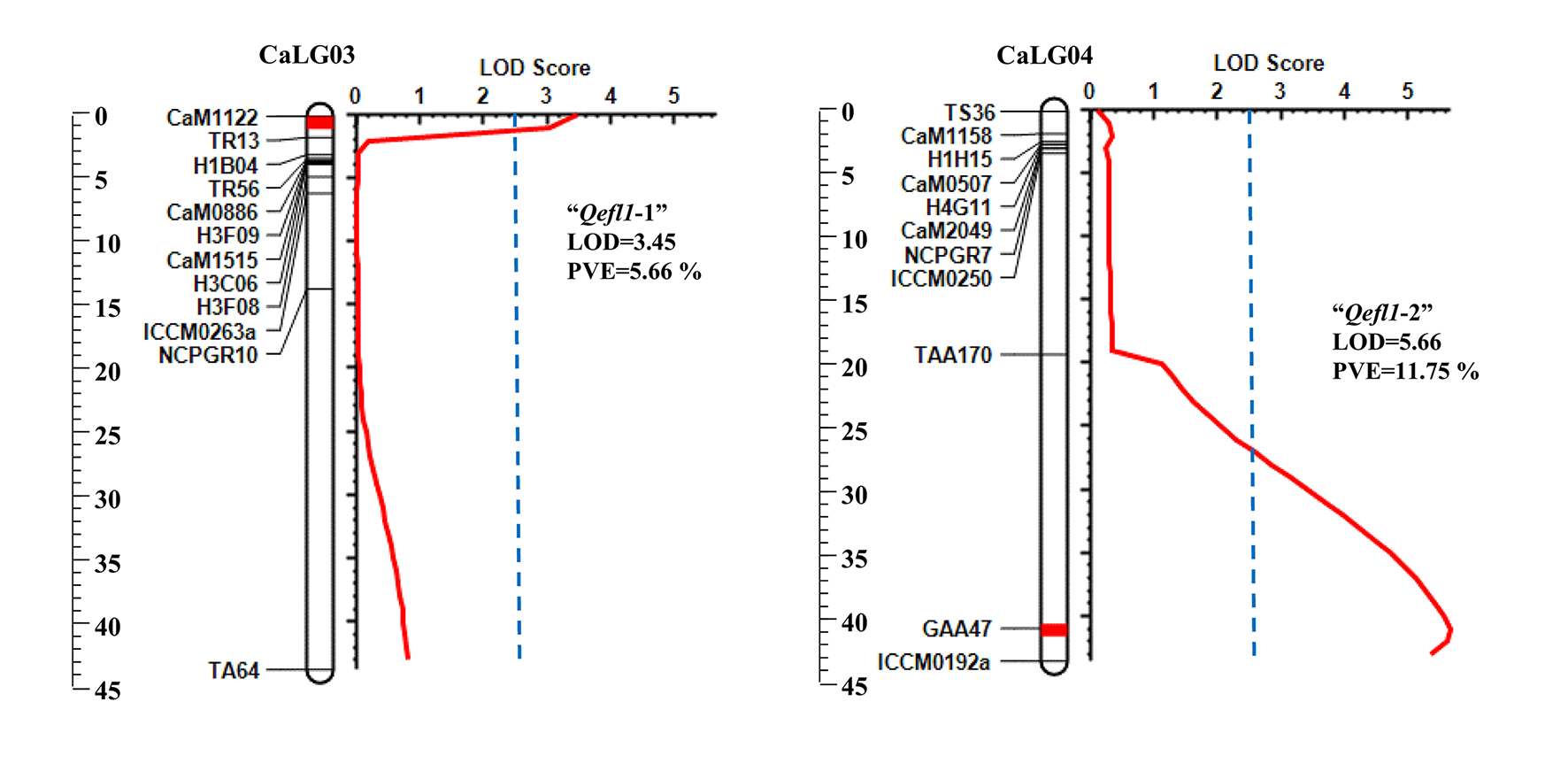

Supplement: Supplementary Figure 5 — QTLs identified for flowering time in the cross ICCV 96029 × CDC Frontier. A major QTL on CaLG04 and a minor QTL on CaLG03 were identified for flowering time in this cross. [file Image5.TIF]

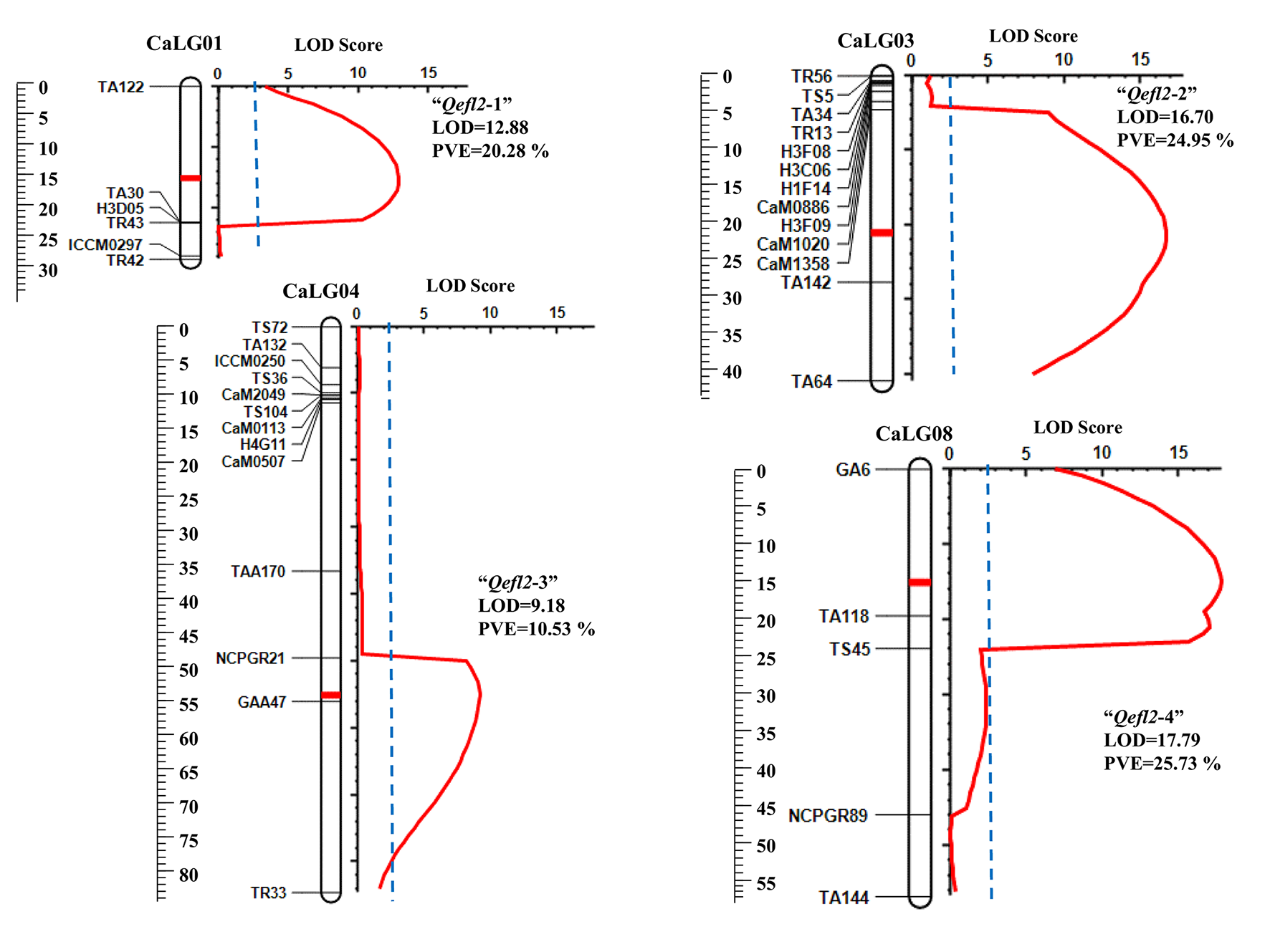

Supplement: Supplementary Figure 6 — QTLs identified for flowering time in the cross ICC 5810 × CDC Frontier. A major QTL each on CaLG01, CaLG03, CaLG04, and CaLG08 were identified for flowering time in this cross. [file Image6.TIF]

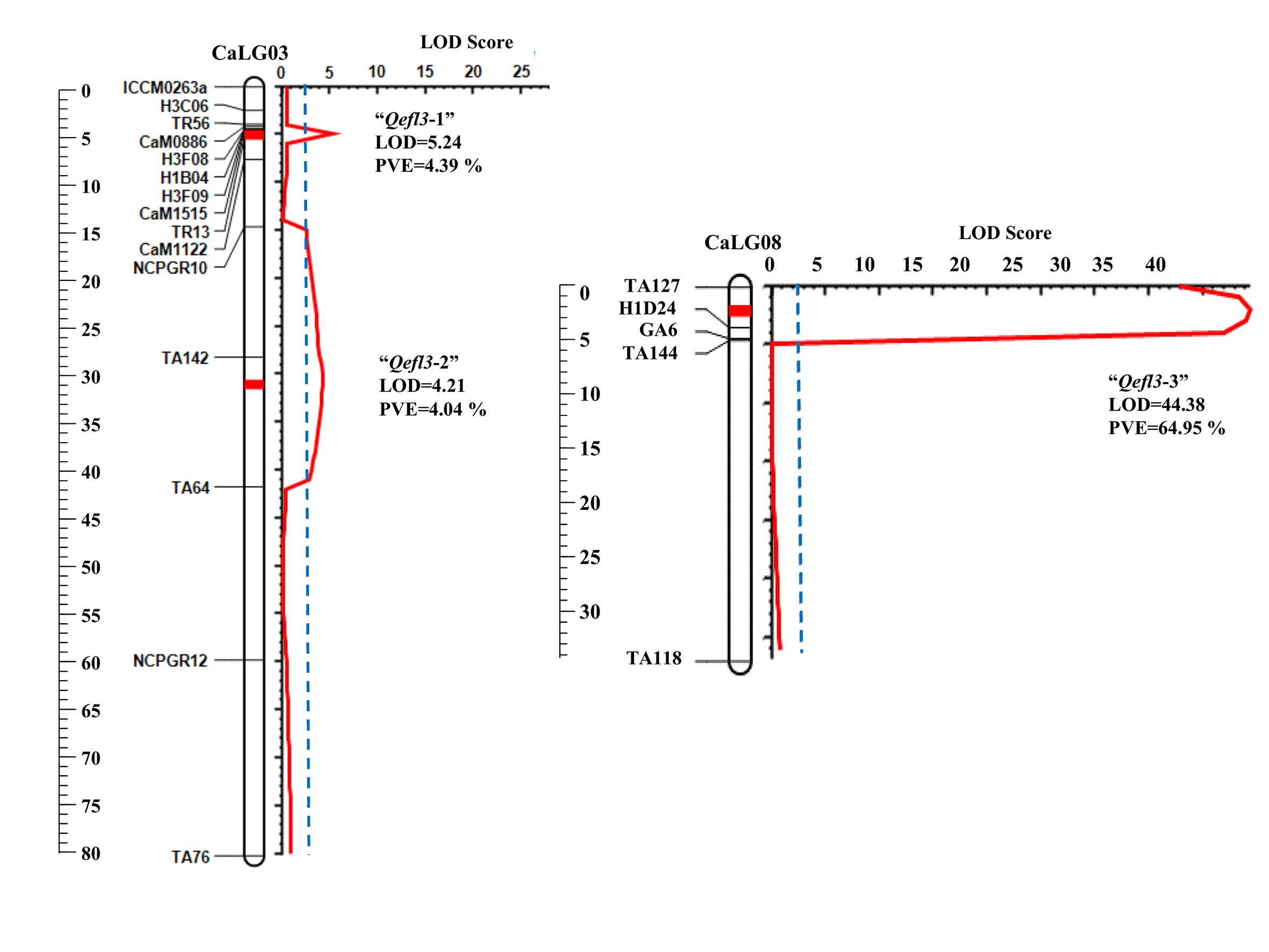

Supplement: Supplementary Figure 7 — QTLs identified for flowering time in the cross BGD 132 × CDC Frontier. A major QTL on CaLG08 and two minor QTLs on CaLG03 were identified for flowering time in this cross. [file Image7.TIF]
