# Supplementary material for: Molecular Mapping of Flowering Time Major Genes and QTLs in Chickpea (Cicer arietinum L.)
Source: Front Plant Sci. 2017 Jul 6;8:1140. doi: 10.3389/fpls.2017.01140 (PMC5498527; doi:10.3389/fpls.2017.01140)
Supplement: Supplementary Table 1 — Mean and range of variation of flowering time and maturity in parental lines. [file Table1.DOCX]

**Supplementary Table 1. Mean and range of variation of flowering time and maturity in parental lines**

| **Sl. No.** | **Parents** | **N** | **Days to first flower** | |  | **Days to maturity** | |
| --- | --- | --- | --- | --- | --- | --- | --- |
|  |  |  | **Mean ± SE** | **Range** |  | **Mean ± SE** | **Range** |
| 1 | ICCV 96029 | 20 | 26.85 ± 0.21 | 25-28 |  | 75.55 ± 0.30 | 75-81 |
| 2 | ICC 5810 | 20 | 28.35 ± 0.15 | 28-30 |  | 77.25 ± 0.16 | 77-80 |
| 3 | BGD 132 | 20 | 28.55 ± 0.11 | 28-29 |  | 78.95 ± 0.23 | 78-83 |
| 4 | ICC 16641 | 20 | 29.00 ± 0.00 | 29-29 |  | 79.00 ± 0.00 | 79-79 |
| 5 | CDC Frontier | 20 | 66.90 ± 0.25 | 65-68 |  | 108.15 ± 0.59 | 104-112 |
