# Supplementary material for: Molecular Mapping of Flowering Time Major Genes and QTLs in Chickpea (Cicer arietinum L.)
Source: Front Plant Sci. 2017 Jul 6;8:1140. doi: 10.3389/fpls.2017.01140 (PMC5498527; doi:10.3389/fpls.2017.01140)
Supplement: Supplementary Table 2 — Mean and range of variation of flowering time and maturity in F1s and F2 populations. [file Table2.DOCX]

**Supplementary Table 2. Mean and range of variation of flowering time and maturity in F_1_s and F_2_ populations**

|  |  | **F_1_** | | | | |  | **F_2_** | | | | |
| --- | --- | --- | --- | --- | --- | --- | --- | --- | --- | --- | --- | --- |
| **Sl. No.** | **Cross** | **N** | **Days to first flower** | | **Days to maturity** | |  | **N** | **Days to first flower** | | **Days to maturity** | |
|  |  |  | **Mean ± SE** | **Range** | **Mean ± SE** | **Range** |  |  | **Mean ± SE** | **Range** | **Mean ± SE** | **Range** |
| 1 | ICCV 96029 × CDC Frontier | 20 | 61.20 ± 0.33 | 59-63 | 92.95 ± 0.46 | 90-95 |  | 190 | 48.89 ± 0.80 | 25-77 | 89.63 ± 0.56 | 73-116 |
| 2 | ICC 5810 × CDC Frontier | 20 | 54.15 ± 0.19 | 53-55 | 89.75 ± 0.76 | 84-95 |  | 190 | 49.47 ± 0.69 | 27-76 | 89.92 ± 0.49 | 76-113 |
| 3 | BGD 132 × CDC Frontier | 20 | 53.30 ± 0.31 | 51-55 | 94.50 ± 0.53 | 91-98 |  | 190 | 49.04 ± 0.92 | 26-75 | 92.19 ± 0.66 | 73-120 |
| 4 | ICC 16641 × CDC Frontier | 20 | 60.80 ± 0.39 | 59-64 | 94.65 ± 0.60 | 90-99 |  | 146 | 52.93 ± 1.26 | 26-82 | 91.83 ± 0.92 | 72-120 |
