# Supplementary material for: Molecular Mapping of Flowering Time Major Genes and QTLs in Chickpea (Cicer arietinum L.)
Source: Front Plant Sci. 2017 Jul 6;8:1140. doi: 10.3389/fpls.2017.01140 (PMC5498527; doi:10.3389/fpls.2017.01140)
Supplement: Supplementary Table 3 — Segregation of flowering time in F3 progenies of four chickpea crosses. [file Table3.DOCX]

**Supplementary Table 3. Segregation of flowering time in F_3_ progenies of four chickpea crosses**

| **Sl. No.** | **Cross** | **Phenotypic class** | **No. of progeny tested** | **Observed** | |  | **Expected** | | **Ratio tested** | **χ^2^** | **P-value*** |
| --- | --- | --- | --- | --- | --- | --- | --- | --- | --- | --- | --- |
|  |  |  |  | **Segregating** | **Non-segregating** |  | **Segregating** | **Non-segregating** |  |  |  |
| 1 | ICCV 96029 × CDC Frontier | Late | 127 | 87 | 40 |  | 84.6 | 42.3 | 2:1 | 0.19 | 0.7-0.5 |
|  |  | Early | 37 | 0 | 37 |  | 0 | 37 | 0:1 | 0 | 1.0 |
| 2 | ICC 5810 × CDC Frontier | Late | 103 | 87 | 16 |  | 91.5 | 11.4 | 8:1 | 2.04 | 0.2-0.1 |
|  |  | Early | 71 | 44 | 27 |  | 40.5 | 30.4 | 4:3 | 0.67 | 0.5-0.3 |
| 3 | BGD 132 × CDC Frontier | Late | 138 | 94 | 44 |  | 92 | 46 | 2:1 | 0.13 | 0.8-0.7 |
|  |  | Early | 44 | 0 | 44 |  | 0 | 44 | 0:1 | 0 | 1.0 |
| 4 | ICC 16641 × CDC Frontier | Late | 77 | 54 | 23 |  | 51.3 | 25.6 | 2:1 | 0.41 | 0.7-0.5 |
|  |  | Early | 25 | 0 | 25 |  | 0 | 25 | 0:1 | 0 | 1.0 |

*Null hypothesis of the test is that progeny segregate in the ratios tested. If the p-value (probability) is less than or equal to 0.05, then reject the null hypothesis. Otherwise one fails to reject the null hypothesis.
