# Supplementary material for: Molecular Mapping of Flowering Time Major Genes and QTLs in Chickpea (Cicer arietinum L.)
Source: Front Plant Sci. 2017 Jul 6;8:1140. doi: 10.3389/fpls.2017.01140 (PMC5498527; doi:10.3389/fpls.2017.01140)
Supplement: Supplementary Table 4 — Polymorphism status of SSR markers between parental lines of four intraspecific mapping populations. [file Table4.DOCX]

**Supplementary Table 4. Polymorphism status of SSR markers between parental lines of four intraspecific mapping populations**

| **Markers** | **No. of markers screened** | **ICCV 96029 × CDC Frontier** | | **ICC 5810 × CDC Frontier** | | **BGD 132 × CDC Frontier** | | **ICC 16641 × CDC Frontier** | |
| --- | --- | --- | --- | --- | --- | --- | --- | --- | --- |
|  |  | **No. of polymorphic markers** | **Polymorphism (%)** | **No. of polymorphic markers** | **Polymorphism (%)** | **No. of polymorphic markers** | **Polymorphism (%)** | **No. of polymorphic markers** | **Polymorphism (%)** |
|  |  |  |  |  |  |  |  |  |  |
|  |  |  |  |  |  |  |  |  |  |
| CaM-series | 146 | 18 | 12.33 | 18 | 12.33 | 16 | 10.96 | 14 | 9.59 |
| H-series | 57 | 16 | 28.07 | 13 | 22.81 | 17 | 29.82 | 16 | 28.07 |
| ICCM-series | 124 | 8 | 6.45 | 5 | 4.03 | 5 | 4.03 | 7 | 5.65 |
| NCPGR-series | 10 | 5 | 50.0 | 3 | 30.0 | 4 | 40.0 | 5 | 50.0 |
| Winter-series | 135 | 53 | 40.0 | 56 | 41.48 | 48 | 35.56 | 51 | 37.78 |
| **Total** | **472** | **100** | **21.40** | **95** | **20.13** | **90** | **19.07** | **93** | **19.70** |
