# Supplementary material for: Molecular Mapping of Flowering Time Major Genes and QTLs in Chickpea (Cicer arietinum L.)
Source: Front Plant Sci. 2017 Jul 6;8:1140. doi: 10.3389/fpls.2017.01140 (PMC5498527; doi:10.3389/fpls.2017.01140)
Supplement: Supplementary Table 6 — Coordinates for the flowering time QTLs identified in four chickpea crosses. [file Table6.DOCX]

**Supplementary Table 6. Coordinates for the flowering time QTLs identified in four chickpea crosses**

| **Sl. No.** | **Cross** | **QTL** | **CaLG** | **Position (cM)** | **LOD** | **Flanking marker coordinates** | | | |
| --- | --- | --- | --- | --- | --- | --- | --- | --- | --- |
|  |  |  |  |  |  | **Left marker** | **Position (cM)** | **Right Marker** | **Position (cM)** |
| 1 | ICCV 96029 × CDC Frontier | *Qefl1*-1 | 3 | 0.00 | 3.45 | CaM1122 | 0.0 | TR13 | 1.7 |
|  |  | *Qefl1*-2 | 4 | 41.00 | 5.66 | GAA47 | 40.9 | ICCM0192a | 43.5 |
| 2 | ICC 5810 × CDC Frontier | *Qefl2*-1 | 1 | 15.00 | 12.88 | TA122 | 0.0 | TA30 | 22.3 |
|  |  | *Qefl2*-2 | 3 | 21.00 | 16.70 | CaM1358 | 4.5 | TA142 | 27.6 |
|  |  | *Qefl2*-3 | 4 | 55.00 | 9.18 | NCPGR21 | 49.4 | GAA47 | 55.9 |
|  |  | *Qefl2*-4 | 8 | 15.00 | 17.79 | GA6 | 0.0 | TA118 | 19.4 |
| 3 | BGD 132 × CDC Frontier | *Qefl3*-1 | 3 | 5.00 | 5.24 | CaM1515 | 4.6 | TR13 | 5.2 |
|  |  | *Qefl3*-2 | 3 | 31.00 | 4.21 | TA142 | 28.2 | TA64 | 41.7 |
|  |  | *Qefl3*-3 | 8 | 2.00 | 44.38 | TA127 | 0.0 | H1D24 | 3.4 |
| 4 | ICC 16641 × CDC Frontier | *Qefl4*-1 | 6 | 9.00 | 55.60 | TA14 | 0.0 | TR44 | 17.4 |
